# Author Correction: Satellitome comparison of two oedipodine grasshoppers highlights the contingent nature of satellite DNA evolution

**DOI:** 10.1186/s12915-022-01260-z

**Published:** 2022-03-23

**Authors:** Juan Pedro M. Camacho, Josefa Cabrero, María Dolores López-León, María Martín-Peciña, Francisco Perfectti, Manuel A. Garrido-Ramos, Francisco J. Ruiz-Ruano

**Affiliations:** 1 Departamento de Genética, Universidad de Granada, 18071 Granada, Spain; 2 Research Unit Modeling Nature, Universidad de Granada, Granada, Spain; 3 Department of Organismal Biology – Systematic Biology, Evolutionary Biology Centre, Uppsala University, SE-752 36 Uppsala, Sweden; 4 School of Biological Sciences, University of East Anglia, Norwich Research Park, Norwich, NR4 7TU UK

**Correction to: BMC Biology 20, 1–24 (2021)**

**https://doi.org/10.1186/s12915-021-01216-9**

The original article contained an error by the production team whereby Figs. 1–3 were mistakenly inverted causing a mismatch with the figures’ respective captions. The original article has since been corrected to match each figure to the correct caption.

