# Supplementary figures and images for: Satellitome comparison of two oedipodine grasshoppers highlights the contingent nature of satellite DNA evolution
Source: BMC Biol. 2022 Feb 7;20:36. doi: 10.1186/s12915-021-01216-9 (PMC8822648; doi:10.1186/s12915-021-01216-9)

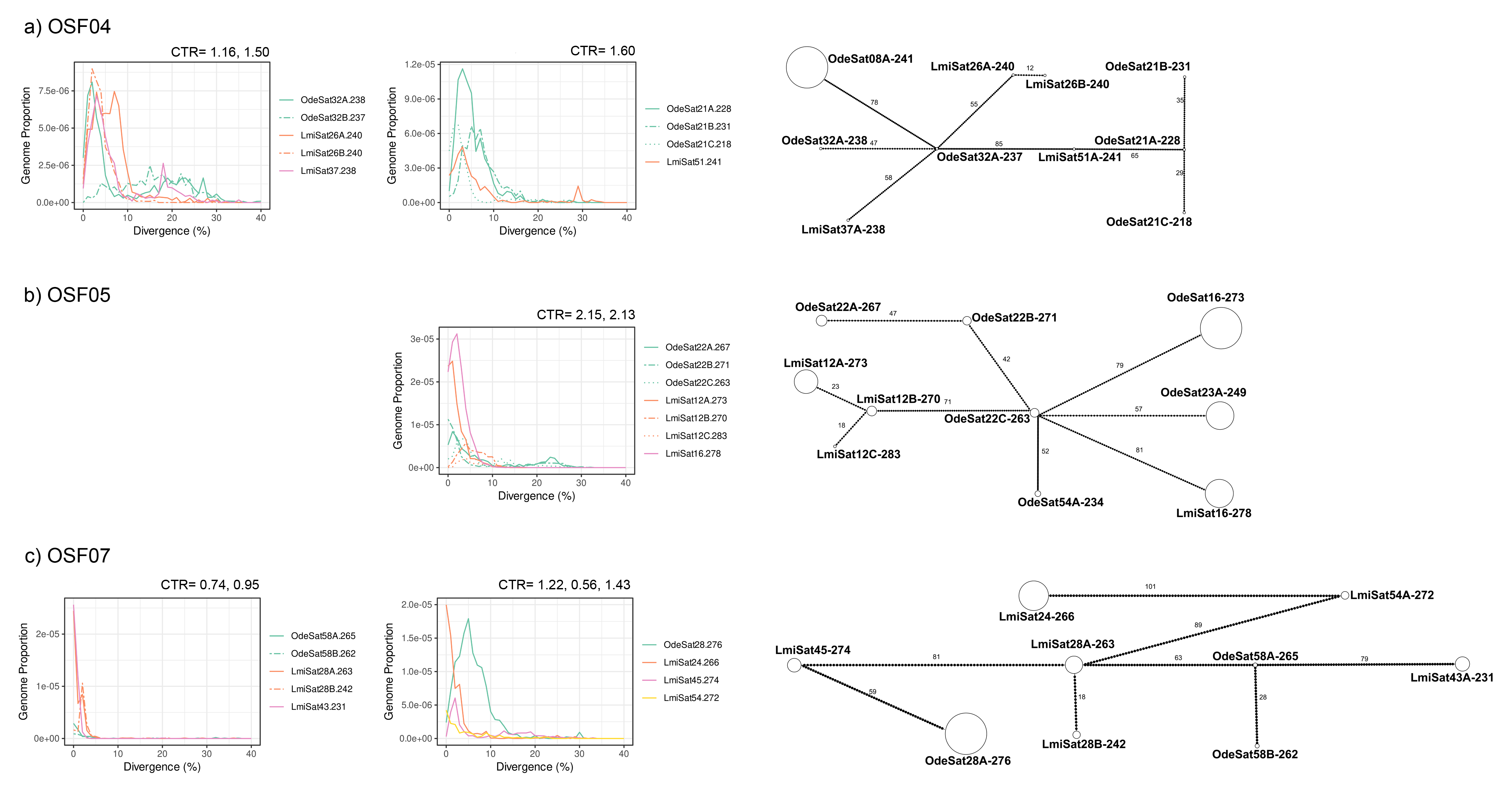

Supplement: Supplementary file 2 — Additional file 2: Figure S1. Repeat landscape (RL) and minimum spanning tree (MST) of three orthologous superfamilies of satellite DNA in O. decorus and L. migratoria (OSF04, OSF05 and OSF07). a) RLs showed that OSF04 showed large peaks of amplification in both species but CTR values ranged between 1.16 and 1.6, presumably due to the incomplete library sorting (ILibS) evidenced by the MST (note how OdeSat32A and LmiSat51A connect with both species' sequences). b) OSF05 showed high CTR values, large amplification peaks in both species and ILibS for only OdeSat22C, which was the only sequence connected with sequences from both species. c) OSF07 showed the lowest CTR values and showed very small amplification peaks for OdeSat58 (green curves in the RL on the left) and higher ILibS, with three sequences being connected with both species' sequences (LmiSat45-274, LmiSat28A-263 and OdeSat58A-265). [file 12915_2021_1216_MOESM2_ESM.tif]
